# Perceptual Training in Beach Volleyball Defence: Different Effects of Gaze-Path Cueing on Gaze and Decision-Making

**DOI:** 10.3389/fpsyg.2015.01834

**Published:** 2015-12-01

**Authors:** André Klostermann, Christian Vater, Ralf Kredel, Ernst-Joachim Hossner

**Affiliations:** Institute of Sport Science, University of BernBern, Switzerland

**Keywords:** perceptual-cognitive skills, anticipation, gaze behavior, eye tracking, sport performance

## Abstract

For perceptual-cognitive skill training, a variety of intervention methods has been proposed, including the so-called “color-cueing method” which aims on superior gaze-path learning by applying visual markers. However, recent findings challenge this method, especially, with regards to its actual effects on gaze behavior. Consequently, after a preparatory study on the identification of appropriate visual cues for life-size displays, a perceptual-training experiment on decision-making in beach volleyball was conducted, contrasting two cueing interventions (functional vs. dysfunctional gaze path) with a conservative control condition (anticipation-related instructions). Gaze analyses revealed learning effects for the dysfunctional group only. Regarding decision-making, all groups showed enhanced performance with largest improvements for the control group followed by the functional and the dysfunctional group. Hence, the results confirm cueing effects on gaze behavior, but they also question its benefit for enhancing decision-making. However, before completely denying the method’s value, optimisations should be checked regarding, for instance, cueing-pattern characteristics and gaze-related feedback.

## Introduction

In sport games, players have to perform under severe time constraints that require decisions to be made within very short time frames. To successfully solve those tasks, it is often demanded to predict the opponents’ future actions to compensate processing delays in the sensorimotor system. Accordingly, with regards to these predictive visual-cognitive skills, it is not surprising that expert athletes outperform their less-experienced counterparts (for overviews, Starkes and Ericsson, 2003; Mann et al., 2007). In particular, it is repeatedly reported in anticipation research that experts are better able to extract crucial information from the opponents’ kinematics at a very early stage, thereby increasing the chance to correctly predict the outcome. In this regard, Williams et al. (2009) studied anticipation in skilled vs. less-skilled tennis players. By using point-light displays, they manipulated the dynamics of the movements of the presented opposing tennis player. Compared with a baseline condition, an impaired anticipation performance in skilled players was revealed when both proximal and distal kinematic features were manipulated, whereas for the less-skilled players, an impairment was found for the distal manipulation only. As, in tennis strokes, distal movements define the action outcome comparatively late in the execution phase, the authors concluded that experts’ anticipation skill is characterized by a more global gaze strategy relying also on early kinematic information in the decision-making process. In contrast, less-skilled players seem to utilize late kinematic cues only and to rely exclusively on end-point trajectory information.

At this point, from an applied perspective, the questions arise whether such anticipation skills can be trained, and if so, how respective interventions shall be optimally structured. When pertaining to the first question, Haskins (1965) already revealed improvements in anticipating opponents’ tennis returns when perceptually training tennis players with respective video sequences. Over the following decades, the functionality of anticipation skill training has been further empirically underpinned by introducing control and placebo groups. For example, Abernethy et al. (1999) showed large improvements from pre- to post-test in the ability to anticipate tennis strokes for a perceptual training group, whereas a placebo group, whose participants were provided with a comparable amount of motor practice, did not improve at all.

Recently, research has concentrated on the effectivity of training instructions. In this regard, numerous approaches have been tested (for an overview, e.g., Williams et al., 2011) that can be divided into more explicit and more implicit methods. Explicit methods focus on imparting strategies, directly allowing to acquire if-then rules on critical action sequences as well as to become aware of information-rich areas and critical cues, particularly kinematic cues. These methods are provided either in the form of detailed instructions (e.g., Williams et al., 2003) or in combination with visual cues that highlight the respective cue on the respective training footage (e.g., Ryu et al., 2013). In comparison, implicit methods aim to optimize anticipatory skills without directly imparting instructions but by promoting self-learning processes. These include approaches such as introducing dual-task paradigms in which learners’ attention gets directed toward a secondary task while the primary task is learned (e.g., Farrow and Abernethy, 2002), (guided) discovery approaches that encourage the learner to identify (specific) relations and regularities in anticipatory tasks (e.g., Smeeton et al., 2005), and so-called gaze-path-cueing approaches wherein, by the use of (colorized) visual markers, the learner’s gaze is guided to information-rich areas derived from experts’ gaze strategies (e.g., Hagemann et al., 2006).

However, the empirical evidence on the effectiveness of the different methods regarding anticipatory-skill learning for enhancing decision-making is inconsistent. When, for instance, comparing verbal instructions and flicker cueing (a flashing red semi-transparent patch) in a 3-on-2 soccer decision-making task, Cañal-Bruland (2009) revealed shorter decision times for the flicker-cueing trials but no advantages in decision accuracy. Furthermore, Hagemann et al. (2006) investigated the effects of an attention-oriented training (video stimuli with red patches highlighting crucial body regions of the opposing player) with a video training (video stimuli without any manipulations) and a control group (no training) in a badminton anticipation test. Although the attention-oriented training group showed clear advantages when compared with the control group and, beyond, larger improvements from post- to retention test than the video-training group, no differences to the video-training group could be found in the retention test. Finally, Savelsbergh et al. (2010) found superior learning rates in a football-penalty anticipation task for a perceptual-learning group that was trained with edited film clips highlighting the run-up of the penalty taker when compared with the training group that watched the same film clips without highlighted run-up and a control group that received no training between the tests. However, as this superiority comes along with prolonged response times, it is unclear whether the advantage can be attributed to improved anticipation skills or whether the additional use of later information is the decisive factor.

Recently, Abernethy et al. (2012) carried out a comprehensive study of different perceptual-training methods in a handball-goalkeeping anticipation task. Results showed largest improvements in response accuracy for an explicit group receiving if-then rules on the relationship between movement kinematics and shot directions, followed by an implicit group that executed a pair-wise judgment task and a verbal-cueing group that was given the instruction to attend to the throwers’ shoulder. Surprisingly, no improvement was found for the color-cueing group when compared with the control group that did not practice at all. Moreover, verbal reports on cues that participants considered for decision-making revealed that participants of the color-cueing group indicated less rules corresponding to the highlighted cue but increased search for other kinematic cues. Consequently, Abernethy et al. (1999, p. 152) concluded that “any use of color cueing within perceptual training regimes should be done with caution. If color cueing is to be used effectively it will clearly require approaches different to those trialed here.”

These findings add to the rather inconsistent literature sketched above (Hagemann et al., 2006; Cañal-Bruland, 2009; Savelsbergh et al., 2010) so that the question has to be raised whether the application of visual markers results in changes of participants’ gaze behavior or not. This question is crucial in the context at hand as the cueing methods are fundamentally based on the assumption it is the gaze (and by association the attention) that must be guided to crucial areas as, by this gaze shift, the visual attention gets allocated to the respective cues and the processing of the relevant information is (automatically) improved. However, this assumption does not necessarily hold so that empirical research would be needed where the participants’ gaze behavior is independently checked.

When striving for an answer to the resulting research question the application of eye-tracking methods is required. At this point, it comes as a surprise that to date, to our knowledge, only one perceptual-training study on the cueing method has been conducted, which included such a manipulation check, the study conducted by Savelsbergh et al. (2010). They analyzed participants’ gaze behavior in respect to (a-priori) qualitatively categorized search patterns by means of differing fixation sequences (e.g., pattern 5: two separate fixations at the top of the penalty tacker followed by a saccade to the ball area; p. 32). The results showed a positive linear trend toward the trained visual search pattern over the intervention phase for the perceptual-learning group with highlighted film clips. However, the learning rate was rather small (∼10%) and a significant repeated-measures effect from pre- to post-test could not be revealed. Furthermore, the application of a video-based eye-tracking system allows only for collecting low-resolution data (50 Hz in Savelsbergh et al., 2010), making it difficult to accurately capture gaze behavior. Above, data analysis had to be conducted manually so that only a small amount of data could be captured, and the objectivity of the labeling of search patterns seems to be discussable (for more details see Kredel et al., 2015). In addition, participants quite frequently used the search pattern, which had to be learned by the perceptual training group already in the pre-test. Due to this resemblance, participants of the perceptual-training group might have had problems in differentiating between the self-chosen search pattern and the gaze path to be learned. Thus, Savelsbergh et al. (2010) considered a manipulation check to control for their gaze intervention but it seems fair to state that the question whether the gaze-path cueing method has a lasting effect on gaze behavior has not been entirely answered yet.

Consequently, further research seems to be needed on the question whether gaze-path-cueing techniques actually lead to the intended gaze behavior. Furthermore, in this research, the methodological shortcomings addressed above should be overcome. Hence, in the following, a study will be reported that aimed to investigate the effects of gaze-path cueing on learners’ gaze behavior as well as on decision accuracy in a beach volleyball anticipation task. Different from earlier perceptual training studies, a high-frequent, mobile eye-tracking system that allows algorithmic calculations of the participants’ point of gaze and, thereby, for a quantitative and precise check of changes in participants’ gaze behavior was used (for details, see Kredel et al., 2011, 2015). To guarantee that missing effects cannot be attributed to methodological issues (cf. Abernethy et al., 2012), first a preparatory study was conducted, which tested the effects of different visual cue characteristics on gaze behavior and decision accuracy.

## Preparatory Study: Cue Evaluation

**FIGURE 1:**
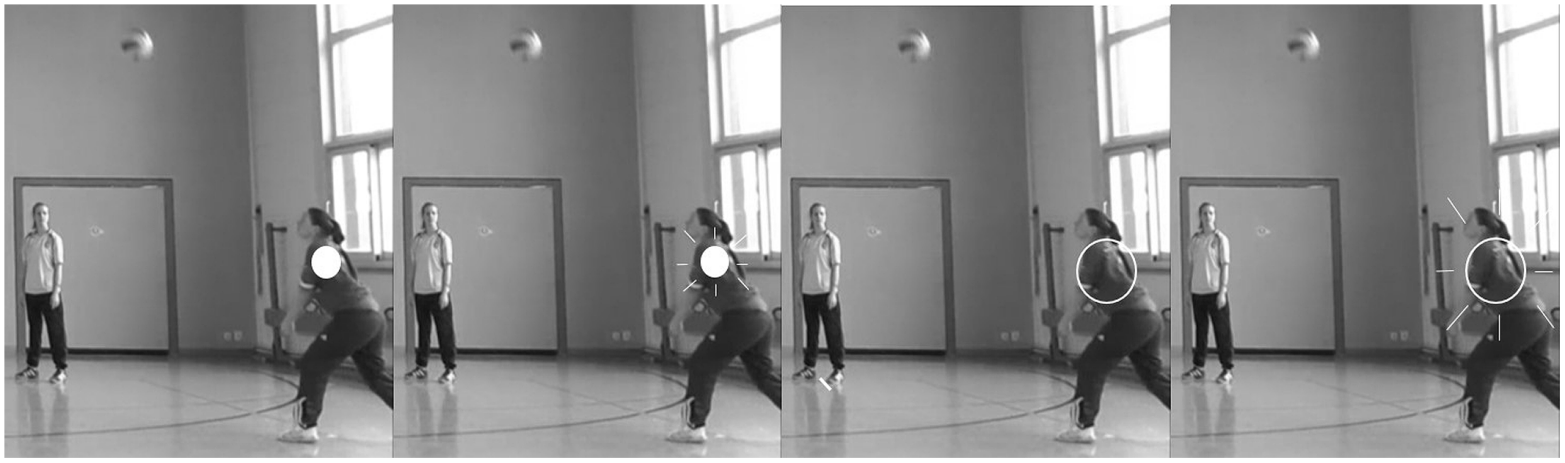
**Visual-cue markers (small/static, small/flashing, large/static, and large/flashing) in a selected frame of the video footage.** Only the two players on the right side of the screen are shown, the player in the front is receiving the ball; original tapes were colored with red markers.

In the preparatory study, decision-making and, especially, gaze behavior were tested as a function of different types of visual cues in a sports-related anticipation task. Furthermore, the aim was to check whether gaze behavior is also drawn by visual cues in life-sized displays as planned to be used in the main study. For this purpose, four groups of 10 sport science students (21 males and 19 females, age: *M* = 21.6 years, *SD* = 1.6 years) watched two times 12 taped (Sony HDR-XR520V, 25 Hz) volleyball-practice situations on a life-sized screen (height: 2.6 m, width: 3.4 m). In these scenes four players (two male and two female) forearm-passed a volleyball three times back and forth that were rectangularly positioned in a gymnastics hall. The participants’ task was to decide three times in each scene by pressing the button as fast and accurate as possible whether either the player in the front or the player in the back would receive the ball. For one of the three decisions in each scene, the receiving player’s hip was marked with a visual cue two frames after the ball had left the proceeding players’ forearms until ball reception. The visual cues between groups differed in sizes (small/1.4° visual angle/filled vs. large/2.8° visual angle/unfilled) and in playback frequencies (static vs. flashing with 5 Hz) (see **Figure 1**). The video scenes were processed using Matlab 2011b and were rendered with MAGIX Video Pro X3.

Participant’s gaze behavior was measured with a VICON-integrated mobile eye-tracking system (EyeSeeCam, 220 Hz) that assesses the vertical and horizontal rotations of both eyes from the pupil and the cornea via infrared reflections. The EyeSeecam system is connected to a MacBook Pro via a 20 m fiber-optic Fire Wire link (GOF-Repeater 800, Unibrain) that is stored in a bum bag so that participants can freely move in the laboratory. The eye-tracker is synchronized by a 10-camera-VICON-T20 system that tracks the three-dimensional (3D) translation and rotation of the participant’s head with three retro-reflective markers attached to the EyeSeeCam at the left, right and top of the camera (cf. Kredel et al., 2015). By doing this, together with the calculated eye rotations, a 3D gaze vector that is updated every 5 ms (Kredel et al., 2011). The accuracy of the eye-tracking system amounts to 0.5° of the visual angle, with a resolution of 0.01° root mean squared error, within 25° of the participant’s field of view. The EyeSeeCam was (re)calibrated at the beginning and in the mid of each test session. Decision-making was gathered by a two-button-response system (1,000 Hz) with one button per hand synchronously recorded with the gaze data by the VICON system. The buttons were covered with colourised tape. The yellow left button had to be pressed if the player in the back (wearing a yellow short) would receive the ball, and the red right button had to be pressed instead if the player in the front (wearing a red shirt) would receive the ball. The approval of the ethics committee of the University Faculty and written informed consent from the participants were obtained in advance. The experiment was undertaken in accordance with the 1964 Declaration of Helsinki.

For each scene, responses with and without gaze-path cues were analyzed. For responses without gaze-path cue, if the first response was cued, the third decision was chosen, and if the second or third response was cued, the respective preceding one was chosen. Thus, all three responses were equally frequently considered in data analysis. Due to problems in data collection for each participant, 1.1% (*SD* = 2.0%) of the trials were not analyzed. As dependent variables, the average moment of decision (relative to the subsequent ball impact by the receiver; ms) and the average decision quality (% correct) were determined. For the gaze data, only correct decisions (*M* = 86.5%, *SD* = 9.0%) were considered. For the valid trials, the average onset, offset, and resulting duration for the first fixation at the receiving player after the preceding pass were calculated (steady gaze point within 1.2° of visual angle for at least 120 ms). All variables were analyzed with a 2 (cueing) × 2 (marker size) × 2 (marker frequency) ANOVA with repeated measures on the first factor. Significant main and interaction effects were further analyzed with follow-up *t*-tests. For decision making it was expected to find earlier and more accurate responses for cued compared to non-cued trials. In addition, it was predicted to find earlier and longer fixations at the receiving player for cued vs. non-cued trials. The preparatory character of the study referred to the marker-related variables and the open question under what conditions the predicted effects would turn out to be maximal.

**FIGURE 2:**
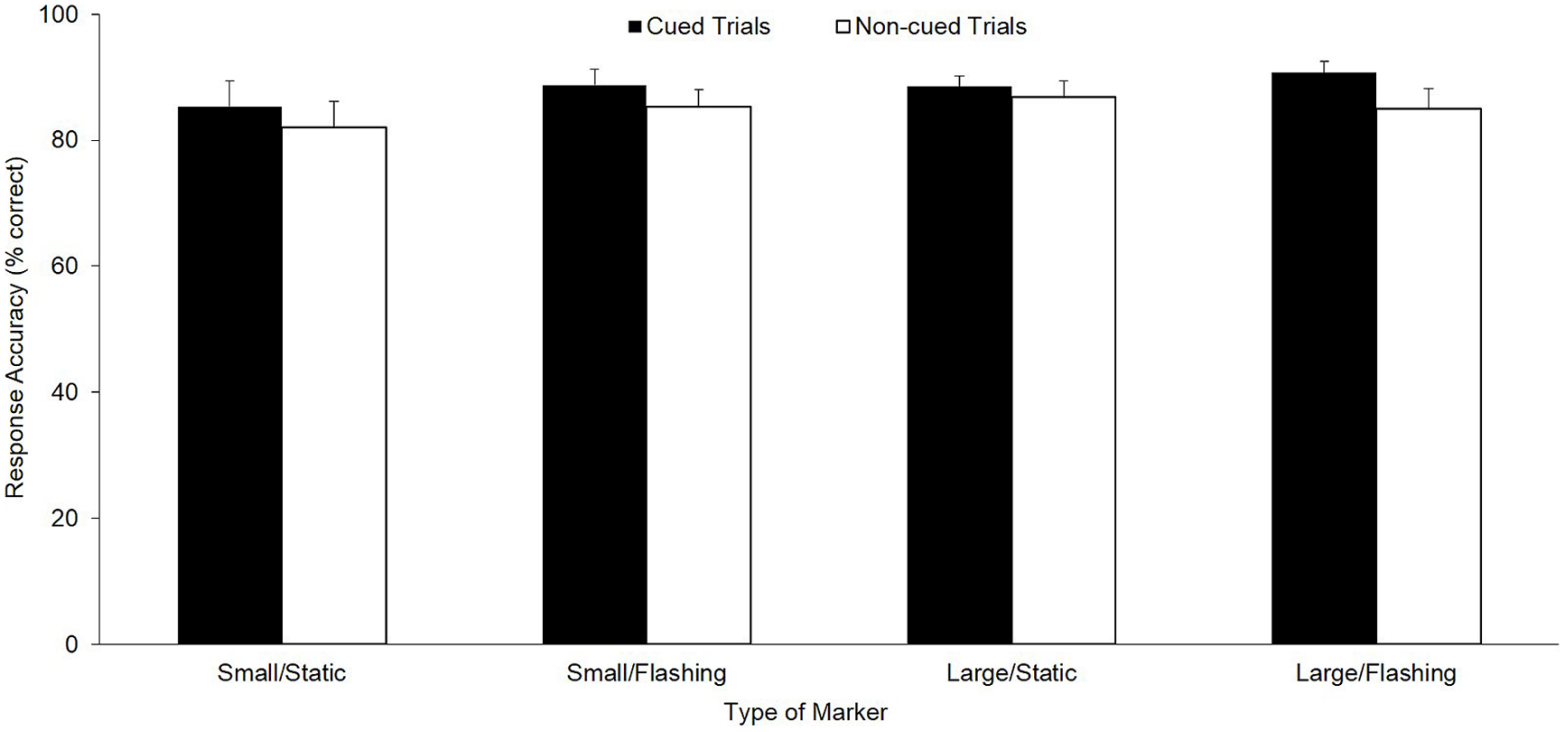
**Response accuracy for decision-making (*M* and *SE*, in % correct) as a function of cued vs. non-cued trials for all four marker types (small/static, small/flashing, large/static, and large/flashing)**.

**FIGURE 3:**
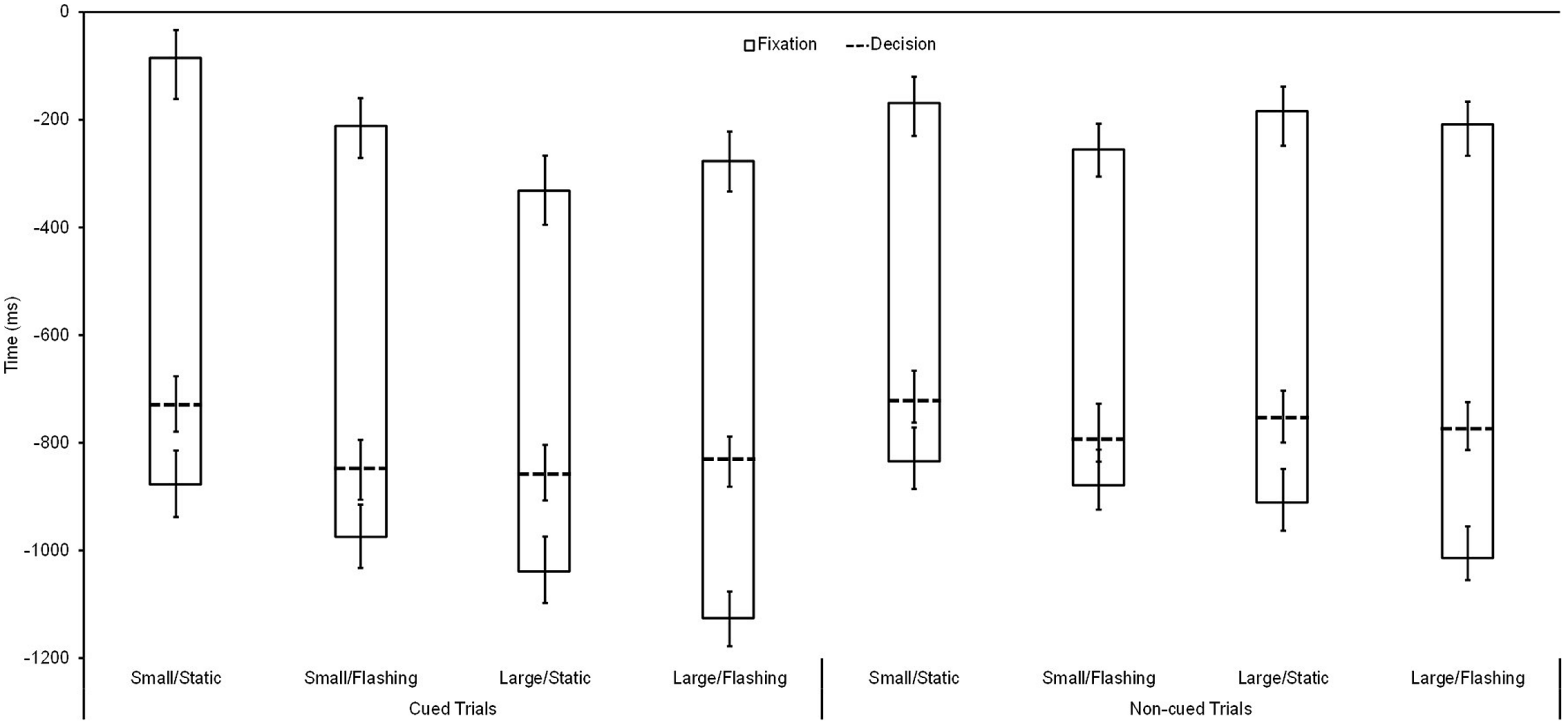
**Fixation onset, fixation offset, and response time (*M* and *SE*, in ms) relative to the moment of subsequent ball reception (=0) as a function of cued vs. non-cued trials for all four marker types (small/static, small/flashing, large/static, and large/flashing)**.

As depicted in **Figure 2**, for response accuracy, a significant main effect was found, *F*(1,36) = 6.93, *p* < 0.01, $\eta_{p}^{2}$ = 0.16, with more accurate decisions in cued (*M* = 88.3%, *SE* = 1.3%) compared with non-cued (*M* = 85.5%, *SE* = 1.5%) trials. Regarding the moment of decision, as illustrated in **Figure 3** as dashed lines, the respective ANOVA revealed a significant main effect for cueing, *F*(1,36) = 48.73, *p* < 0.01, $\eta_{p}^{2}$ = 0.58, as well as a significant three-way interaction, *F*(1,36) = 6.66, *p* = 0.01, $\eta_{p}^{2}$ = 0.16: whereas, with small/static cues, participants decided earlier in cued trials compared with non-cued trails by trend only (*p* = 0.28, $\eta_{p}^{2}$ = 0.16), this difference reached significance for the remaining three cue conditions (small/flashing: *p* < 0.01, $\eta_{p}^{2}$ = 0.80; large/static: *p* < 0.01, $\eta_{p}^{2}$ = 0.70; large/flashing: *p* = 0.02, $\eta_{p}^{2}$ = 0.48). Further significant main and interaction effects for decision-making and gaze behavior were not found (all *p*s > 0.14).

Regarding gaze behavior, which is also depicted in **Figure 3**, for fixation onset, *F*(1,36) = 45.14, *p* < 0.01, $\eta_{p}^{2}$ = 0.56, and duration, *F*(1,36) = 8.62, *p* < 0.01, $\eta_{p}^{2}$ = 0.19, significant main effects for gaze-path cueing were found with earlier and longer fixations for cued than for non-cued trials. Furthermore, considering cued trials only, a significant main effect for size appeared, *F*(1,36) = 8.93, *p* < 0.01, $\eta_{p}^{2}$ = 0.19, with earlier onsets for large than for small cues. Finally, for fixation offset, a (not predicted) cueing × marker size interaction appeared, *F*(1,36) = 6.11, *p* = 0.02, $\eta_{p}^{2}$ = 0.15, with a later offset for small compared to large cues for cued trials only (*p* = 0.01, $\eta_{p}^{2}$ = 0.15) as well as a later offset for the small cue when comparing cued vs. non-cued trials (*p* = 0.05, $\eta_{p}^{2}$ = 0.19). No further significant main effects and interactions were found, particularly, no interactions between cueing and marker type (all *ps* > 0.11).

In sum, the results illustrate that when applying gaze-path cues in a decision-making task, participants decide faster (except for the small/static cue) and more accurately as already reported by Cañal-Bruland (2009). Furthermore, the findings indicate that this superiority seems to be rooted in a specific gaze behavior underpinned by earlier and longer fixations on the cued areas. Above, the absence of any interaction between cueing and type of marker indicates that the marker effects found for the cued trials also seem to be present in the trials in which no cue was presented. This means that, already after a few trials, participants’ gaze behavior was affected by the visual-cue method, which suggests the method’s effectivity.

However, it was also shown that the effect of the gaze-path cuesdepends on its characteristic whereby a clear advantage of large cues was revealed. When comparing large/static and large/flashing markers, analyses deliver differences by-trend only and, beyond this, mixed results. Based on the finding that, by trend again, the earliest moments of decision were found for the large/static markers, in the end, this cue condition was chosen for the perceptual-training interventions in the main study. However, it should be noted that, on the basis of the findings at hand, a preference for large/flashing cues could be substantiated to a more or less equal degree. It has to be acknowledged that the differences found for marker size also can be explained by the distinction filling/no filling; the two factors (size and filling) were not independently manipulated. The small marker was filled since a-priori inspections revealed that the small marker without filling was hardly detectable and presumably would have deteriorated participants’ gaze behavior and decision making.

## Main Study: Perceptual Anticipation Training

The findings of the preparatory study not only point toward the preference for certain markers but also suggest that after a few cue trials only, participants adapt their gaze strategy. Furthermore, this adaptation was accompanied by earlier decisions. On this basis, the question arises whether these effects can also be confirmed in the main study where a real-world situation was investigated, the decision of the defence player in beach volleyball in reaction onto the specific spike of the opposing attacker. This situation was chosen, because a respective expert gaze path could be derived from previous studies (Hossner et al., in preparation; see also Schläppi-Lienhard and Hossner, 2015).

**FIGURE 4:**
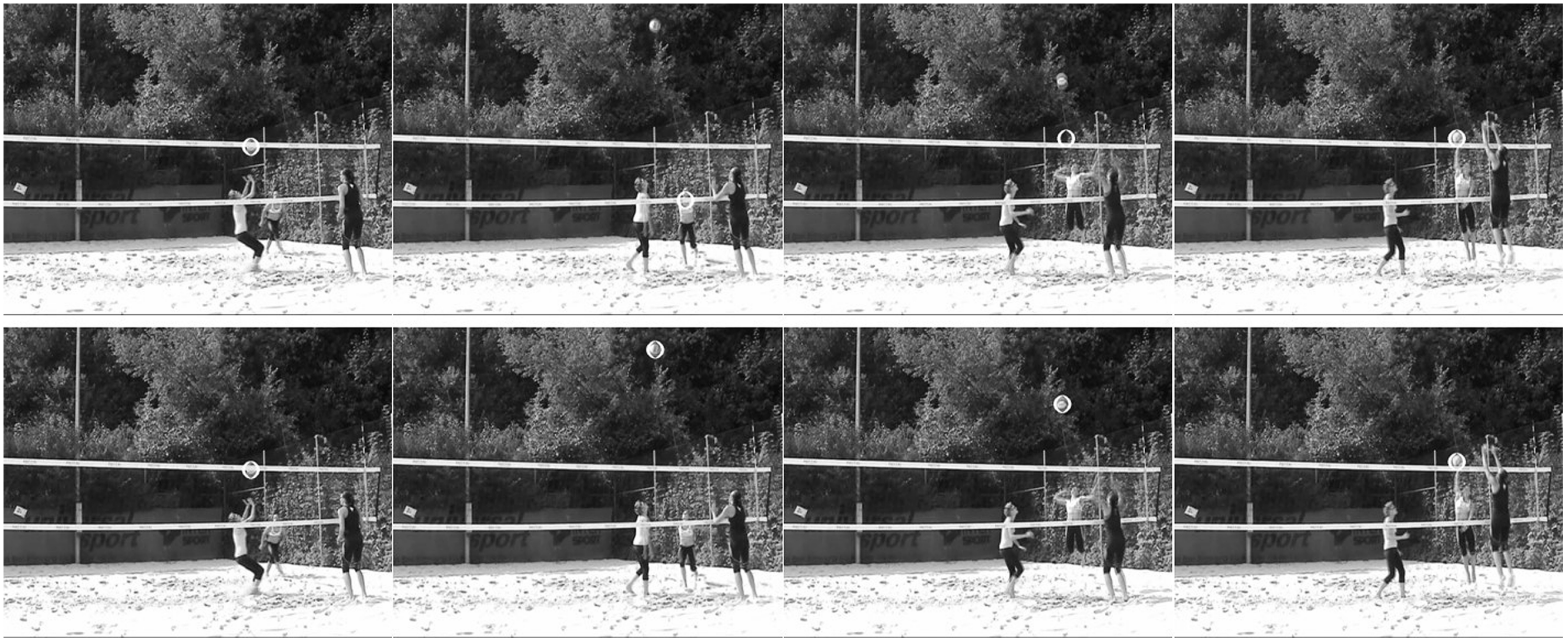
**Functional (top) and dysfunctional (bottom) visual-cue paths at the first frame of phase P0 (set), P1 (run-up), P2 (attack), and the last frame of P2 (attack).** In P0–P2 of the functional path, the ball, the attacker, and the anticipated ball-hand-contact are cued, respectively; in P0–P2 of the dysfunctional path, the ball is always cued. Original tapes were colored with red markers.

In the experiment, as in former perceptual-learning studies, changes of an experimental cue group from pre- to post- and retention test regarding gaze behavior and decision-making needs to be compared with the respective values of a control group. In this respect, a conservative control group was preferred over a control group without any treatment. This means that the participants of the control group watched the same training videos, however, without visual cues, and they were, in addition, asked to identify crucial hints for anticipating the upcoming type of attack. Hence, this intervention can be considered as the fairest possible control as learning advantages of the experimental group would imply that gaze-path cueing actually pays off in comparison to a simple verbal instruction that promotes self-learning. Beyond this, we tried to experimentally disentangle the effect of gaze-path cueing from the learning of an optimal gaze path. For this reason, the design was completed by a third group whose participants were treated with gaze-path cues, which is the same with the case for the experimental group. However, in the experimental group, the expert’s gaze path was highlighted by the markers, whereas in the third group, a gaze path that was significantly deviated from the expert’s path was chosen for gaze-path cueing (see **Figure 4**). Thus, we ended up with a 3-group design with a functional cue group (expert’s gaze path), a dysfunctional cue group (deviating gaze path), and a control group (simple verbal instruction). We expected participants of the cue groups to show the respective gaze path in a post- and retention test, whereas the control group was expected not to change gaze behavior but to constantly show a rather functional than dysfunctional path (Savelsbergh et al., 2010). Furthermore, by assuming that guiding the learners’ gaze to relevant cues promotes anticipatory-skill learning (Hagemann et al., 2006; see also Williams et al., 2002), it was expected to find increased learning for the functional group compared with the dysfunctional and control groups. In addition, based on the expectation that the control group rather would show the expert than the deviating gaze path, increased learning for the control when compared with the dysfunctional group should be found.

### Method

#### Participants

Twenty-two male (age: 22.1 ± 1.5 years) and 23 female (age: 21.4 ± 1.5 years) sport science students received course credits in return for participating in the study. They were assigned to one of three intervention groups on the basis of their pre-test anticipation performance and gaze behavior so that all groups had similar decision-making accuracy and gaze strategies (for details, see Results). Due to technical problems with the eye tracker, one female participant from the dysfunctional group had to be removed from the sample. All participants had self-reported normal or corrected-to-normal vision, and all were unaware of the research question. The approval of the ethics committee of the University Faculty and written informed consent form the participants were obtained in advance. The experiment was undertaken in accordance with the 1964 Declaration of Helsinki.

#### Video Scenes

The video scenes were recorded on a beach volleyball court with a video camera (Sony HDR-XR520V, 25 Hz) that was positioned at the baseline close to the left corner, at a height of 1 m to obtain the perspective of a player defending cross-court smashes with a teammate blocking longline at the right side of the net. In each scene, a standard attacking sequence of the two opposing players was presented which composed of reception, set and attack. Variants were performed neither by the opposing players regarding this sequence nor by the defender’s teammate who always blocked longline so that hard cross-court smashes would be possible. In contrast, the opposing attacker was free to choose one of three options, namely, a hard cross-court smash, a line-shot over the block into the right corner of the court, or a cut shot, which is a diagonally played shot that hits the court very close to the net. Thus, for the defender (who was not shown in the videos), the real-world task would be either to keep his or her position against hard smashes or to run either right-sideward or left-forward to reach line shots and cut shots, respectively.

Only one attacker, an internationally experienced female expert player, was used in the video scenes to allow the acquisition of differentiated knowledge about critical movement components. From 36 raw scenes, only the scenes in which the ball was visible on the video tape during the whole attacking sequence were selected. Finally, four scenes per type of attack (smash, lineshot and cut shot) were selected, that is, 12 scenes in total, which either were presented to the control group or had to be further processed for the gaze-path-cue interventions. Those 12 scenes were presented in the tests as well as in the intervention.

For the cued scenes, either a functional or a dysfunctional gaze path was highlighted by cues (red, large/static, see preparatory study) using a self-written Matlab 2013a routine. In more detail, all scenes were first subdivided into three consecutive phases, P0–P2 (cf., Schläppi-Lienhard and Hossner, 2015), which were defined as follows: P0 (set) from the frame the ball passes the upper edge of the net until two frames after the ball has left the setter’s hands; P1 (run-up) from the end of the previous phase until the initiation of the jump by the upswing of the attacker’s arms; and P2 (attack) from the end of the previous phase until the ball contact by the attacker’s hand. For the functional cue videos, over all three attacking phases, the marker followed the gaze path that had been extracted for expert beach volleyball players in previous studies (Hossner et al., in preparation), namely, the ball over P1, the attacker’s upper body over P2, and the anticipated position of the ball-hand contact by the attacker over P3. In contrast, in the dysfunctional cue videos, the marker highlighted the current position of the ball over all three phases thereby distracting from the attacker’s movement. The resulting different cue paths for the functional and the dysfunctional group are illustrated in **Figure 4**.

After the processing of the raw videos, three sets with 12 scenes each were available: with functional, with dysfunctional, and without cues. For the intervention, these (non-occluded) video scenes were rendered into 12 blocks of 12 video scenes, each in a quasi-randomized order (each block containing each type of attack four times). To prepare the participants of the cue groups for the test situation where they had to pursue the to-be-learned gaze path without cueing, a fading procedure was introduced thereby adopting the logic of the guidance hypothesis that had been introduced by Salmoni et al. (1984) for the problem that learners may become dependent on augmented feedback. Thus, in the cue groups, the frequency of cued scenes was gradually reduced over the treatment phase (blocks 1–4: 100%, blocks 5–8: 67%, blocks 9–12: 33%) and replaced by video scenes without visual cues.

In pre-, post-, and retention tests, only video scenes without cues were presented. In addition, those video scenes were further processed by occluding each scene either seven frames (occ7, i.e., 280 ms) or 1 frame (occ1, i.e., 40 ms) before ball-hand contact of the attacker. Finally, for video footage and participants’ behavior synchronization, audio triggers were added at the beginning of each phase (P0–P2). For each test, the occluded videos were rendered into three blocks of 12 trials each in a quasi-randomized order (each block containing each type of attack four times and each occlusion moment six times) (MAGIX Video Pro X3). Hence, each scene was presented three times in each test.

#### Apparatus

Regarding decision-making on the upcoming type of attack in pre-, post-, and retention tests, participants provided their decisions verbally, and the responses were put down in writing by an experimenter. The gaze behavior was recorded with the ESC system (for details, see preparatory study) that was calibrated at the beginning of each session and, in addition, before each test block of 12 trials if the point of gaze deviated more than 0.5° of the visual angle from one of the points of the calibration grid. Gaze was only recorded in pre-, post-, and retention tests but not for acquisition trials. The data analysis was done with Mathworks MATLAB 2013a. IBM SPSS Statistics 22 was used to conduct statistical analyses.

### Procedure

The study was conducted in the institute’s sensorimotor laboratory. Pre-, post-, and retention tests lasted about 20 min, and the treatment phase lasted about 60 min. The intervention started on average 6.5 days (±2.6 days) after the pre-test, the post-test was conducted after a short break, immediately after the intervention, and the retention test was carried out 1 week later (*M* = 6.8 days ± 0.4 days).

The participants attended individual sessions. After having read the instructions, including an explanation of the different type of attack, they were fitted with the ESC system that was calibrated by consecutively fixating five dots that were displayed in a regular grid with a distance of 8.5° of visual angle between the dots (Kredel et al., 2011). Subsequently, either the test or the intervention session started. In all sessions, the video scenes were displayed at a life-sized screen (height: 2.4 m, width: 3.6 m), and the participants were positioned according to the real defender’s position in the left back-field at a distance of 4.0 m to the screen.

All test sessions started with a warm-up block of 3 (type of attack) times 2 (occlusion) trials followed by three blocks of 12 test trials each (three types of attack × 2 occlusions × 2 repetitions) in which participants had to immediately decide after the occlusion which type of attack they would have to defend. Over the intervention, in 12 blocks of 12 trials each, participants were instructed either to learn the gaze path depicted by the cues (functional and dysfunctional groups) or to get an idea about the presented attack strategies (control group, videos without visual cues) (for details, see Appendix A1). After the retention test, the participants were thanked and debriefed about the objectives of the study.

### Measures

#### Data Processing

Due to technical difficulties in data collection, beyond the exclusion of one participant of the dysfunctional group, 62 trials (=1.3%) in total had to be excluded from further analysis. For calculating distances between participants’ actual gaze and the cue path, first, intersection points between the 3D gaze vector and the screen were calculated, determining the 2D gaze paths in the reference frame of the screen for each trial. Likewise, the digitized 2D visual-cue positions in the videos (25 Hz) were converted into the screen reference frame and up-sampled to 200 Hz by linear interpolation allowing for distance calculations between the gaze vector and the visual-cue position in each frame. The participants’ gaze behavior was considered for further analyses for phases P1 and P2 only as the same cues for both conditions were displayed over P0 (i.e., the ball), and no visual cues were displayed before P0 and after P2.

#### Gaze-Path Index

To obtain single measure, which not only quantifies the distance between the current gaze point and the current position of a cue but also denotes whether the current gaze point is closer to the functional or to the dysfunctional cue path, a gaze-path index (GPI) was computed. To this purpose, for each frame over the relevant video phases, the straight line connecting the current center of the functional and the dysfunctional marker was calculated. Hereafter, the current gaze point was orthogonally projected onto the straight line, and this projection was related to the center of the straight line segment that is defined by the two marker positions. The resulting distance was expressed directionally, in such a way that positive values correspond to a gaze shift in the direction of the dysfunctional and negative values to a gaze shift in the direction of the functional cue path. This scaling had been preferred due to the fact that, over P1 and P2, the dysfunctional path relates to the ball and the functional path relates to the attacker, so that positive values correspond to gaze vectors closer to the upper part of the screen, and negative values to gaze vectors closer to the lower part of the screen. To avoid arbitrary units, the resulting distance vector was then converted from screen-related units (mm) into eye-related differences of visual angle (°) so that a value of, for instance, 0° would have to be interpreted in such a way that the participant’s gaze is currently located exactly in the middle between the functional and the dysfunctional cue position. Finally, for each trial, the frame-by-frame GPI values were averaged over all frames belonging to either P1 or P2, and the resulting phase-related GPI values, dependent on the analysis, were further averaged over types of attack, over occlusion conditions, or over all available 36 trials per pre-, post-, or retention tests.

#### Response Accuracy

The verbal responses were recorded in writing, and the average response accuracy (% correct) was calculated for the pre-, post-, and retention tests (36 trials each) and, if required by the respective analysis, also separately for types of attack (12 trials each) or occlusion conditions (18 trials each).

#### Statistical Analysis

The GPI was subjected to a 3 (group) × 3 (test) × 2 (phase) ANOVA with repeated measures on the last two factors. Response accuracy was analyzed with a 3 (group) × 3 (test) × 3 (type of attack) × 2 (occlusion) ANOVA with repeated measures on the last two factors. Significant main and interaction effects were further analyzed with follow-up *t*-tests. A posteriori effect sizes were computed as partial eta squares ($\eta_{p}^{2}$). Optimal sample sizes were calculated a priori using G^∗^Power 3.1 (see Faul et al., 2009). An optimal sample size of *N* = 45 was revealed on the basis of the expectation of medium effect sizes ($\eta_{p}^{2}$ = 0.06) and settings for the α-level to 0.05 and of the power to 0.95.

### Results

#### Gaze Path Index

**FIGURE 5:**
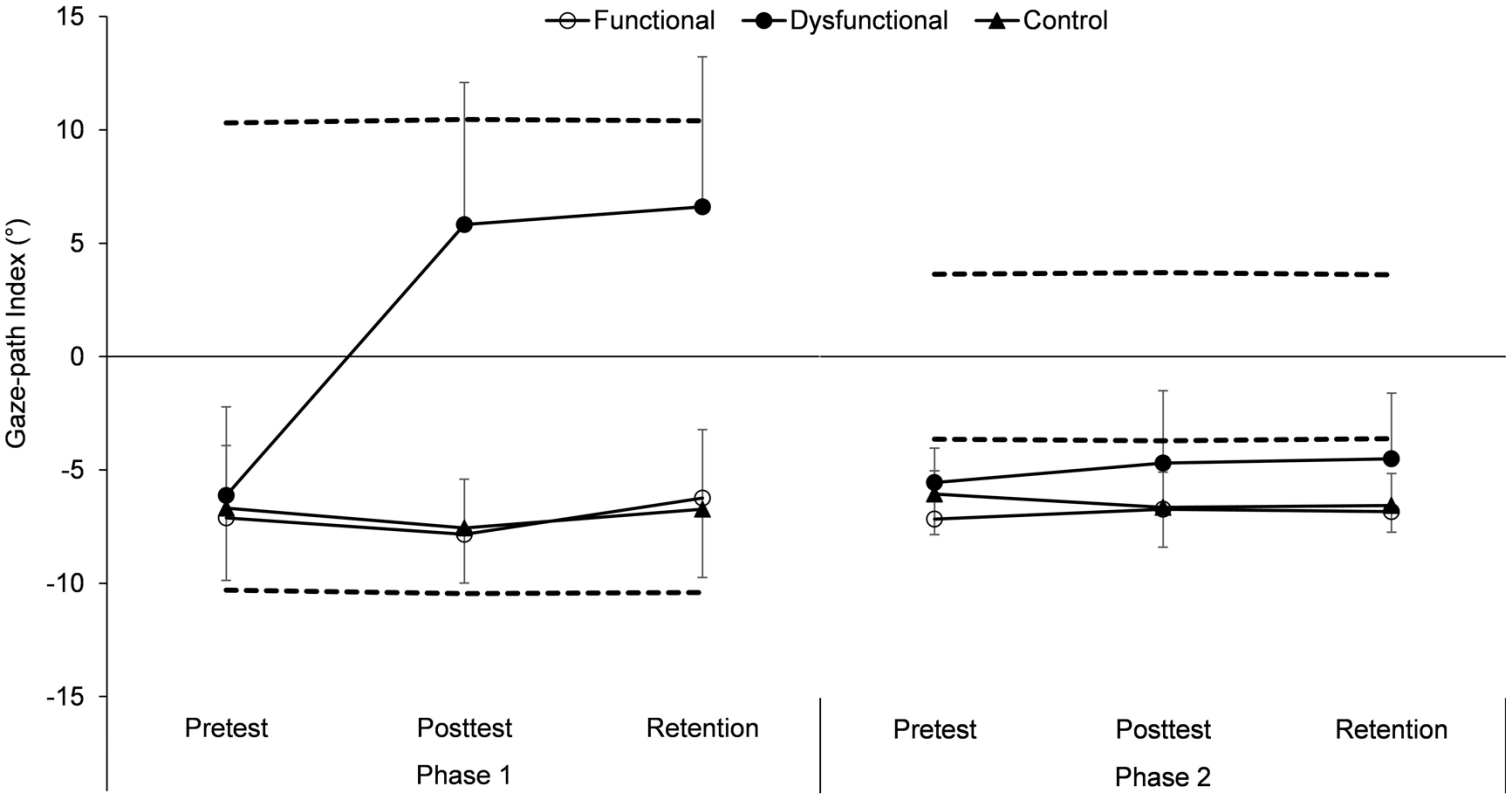
**Gaze-path index (GPI) (*M* and *SE*, in ° visual angle) as a function of group (functional, dysfunctional and control), test (pre-, post-, and retention test), and phase (P1 and P2).** The dashed lines represent the values that were achieved if the gaze perfectly followed either the functional **(lower line)** or the dysfunctional **(upper line)** cue path.

In **Figure 5**, the GPI is depicted as a function of group, test, and phase. The value of 0° on the vertical axis denotes the middle between the functional and dysfunctional cue paths, and the dashed lines correspond to the values a participant would achieve if he or she would exactly follow one of the two cue paths. As it can be taken from the dashed lines, on average, the two functional (negative values) and dysfunctional (positive values) paths are distinguished by a relatively large average distance over P1 (run-up: attacker vs. ball) and a relatively small average distance over P2 (attack: anticipated ball-hand-contact vs. ball). Descriptively, in the pre-test, all GPI values are closer to the functional than to the dysfunctional path. Values below the functional cue path in P2 denote that from jump initiation until ball-hand contact by the attacker participants tend to direct their gaze more to the attacker than to the point of the anticipated ball-hand contact, which is definitive for the gaze path that had been considered a priori as functional.

Inferential-statistically, besides main effects for group, *F*(2,41) = 31.44, *p* < 0.01, $\eta_{p}^{2}$ = 0.61, test, *F*(2,82) = 15.78, *p* < 0.01, $\eta_{p}^{2}$ = 0.28, and phase, *F*(1,41) = 24.19, *p* < 0.01, $\eta_{p}^{2}$ = 0.37, as well as significant two-way interactions for group × test, *F*(4,82) = 16.82, *p* < 0.01, $\eta_{p}^{2}$ = 0.45, group × phase, *F*(2,41) = 33.36, *p* < 0.01, $\eta_{p}^{2}$ = 0.61, and test × phase, *F*(2,82) = 35.34, *p* < 0.01, $\eta_{p}^{2}$ = 0.46, the ANOVA revealed a significant three-way interaction group × test × phase, *F*(4,82) = 34.89, *p* < 0.01, $\eta_{p}^{2}$ = 0.63. When analyzing this interaction, in the pre-test, neither a main effect for group, *F*(2,41) = 1.03, *p* = 0.36, $\eta_{p}^{2}$ = 0.04, nor the group × phase interaction, *F*(2,41) = 0.32, *p* = 0.73, $\eta_{p}^{2}$ = 0.01, were significant, elucidating that all intervention groups started with a similar gaze strategy. However, in the post-, as well as in the retention test, significant main effects for group [post-test: *F*(2,41) = 37.36, *p* < 0.01, $\eta_{p}^{2}$ = 0.65; retention test: *F*(2,41) = 32.37, *p* < 0.01, $\eta_{p}^{2}$ = 0.61] and significant group × phase interactions [post-test: *F*(2,41) = 56.02, *p* < 0.01, $\eta_{p}^{2}$ = 0.73; retention test: *F*(2,41) = 31.69, *p* < 0.01, $\eta_{p}^{2}$ = 0.61] were found. These interactions can be explained in such a way that, in both phases and tests, the dysfunctional group showed at least descriptively [post-test/P2, dysfunctional vs. control: *t*(27) = 1.99, *p* = 0.06, $\eta_{p}^{2}$ = 0.13], but in most cases significantly, a different gaze strategy compared with the functional and the control groups (all *p*s < 0.05), whereas no differences were evident between the functional and the control groups (all *p*s > 0.63). Finally, an additionally calculated *post hoc* ANOVA on the pre-, post-, and retention test results of the functional and the control groups revealed no significant effects (all *p*s > 0.08, all 1-β > 0.06). Hence, summing up, it can be said that (1) the three groups did not differ from each other in the pre-test, that (2) the participants of the functional and the control group did not change their gaze behaviors as a consequence of their specific treatment, and that (3) the dysfunctional group considerably adapted their gaze in the direction of the dysfunctional cue path.

#### Response Accuracy

For response accuracy, significant main effects for type of attack, *F*(1,41) = 13.44, *p* < 0.01, $\eta_{p}^{2}$ = 0.25, occlusion, *F*(1,41) = 114.52, *p* < 0.01, $\eta_{p}^{2}$ = 0.74, as well as a significant test × occlusion interaction, *F*(2,82) = 4.97, *p* < 0.05, $\eta_{p}^{2}$ = 0.11, were found. For cut shots, decision accuracy was inferior compared to hard-cross smash and line-shots (all *p*s < 0.01, all $\eta_{p}^{2}$ > 0.26) which did not significantly differ (*p* = 0.21, $\eta_{p}^{2}$ = 0.04). In addition, participants decided more accurate in occ1 (*M* = 73.8%, *SD* = 17.5%) than in occ7 (*M* = 57.9%, *SD* = 18.4%), which replicates the classical finding that – independent of expertise –later information in the opponents movement pattern allow more accurate predictions of the outcome. Beyond, this difference changed over time with the highest difference in the pre-test (20.9%), followed by the retention (15.0%) and the post-test (11.6%), indicating that with increasing experience the participants were better able also to use early anticipatory information for decision-making. As no significant three-way interactions (all *p*s > 0.49, all $\eta_{p}^{2}$ < 0.04) and no significant four-way interaction appeared, *F*(8,164) = 1.84, *p* = 0.07, $\eta_{p}^{2}$ = 0.08, further analyses on group-difference could be conducted with the mean of the two occlusion conditions.

**FIGURE 6:**
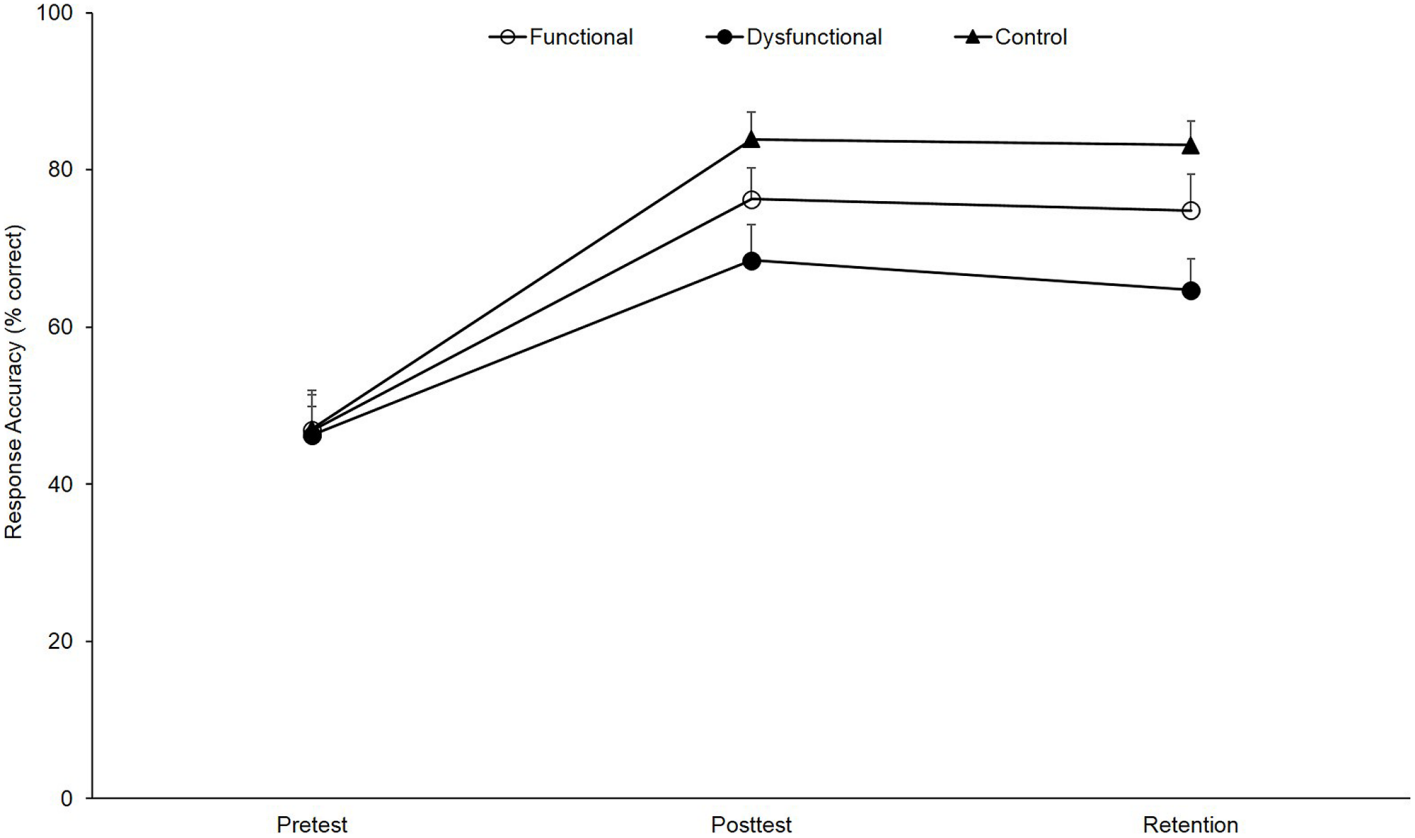
**Response accuracy (*M* and *SE*, in % correct) as a function of group (functional, dysfunctional, and control) and test (pre-test, post-test, and retention)**.

As depicted in **Figure 6**, the respective ANOVA revealed a significant main effect for test, *F*(2,82) = 95.96, *p* < 0.01, $\eta_{p}^{2}$ = 0.70, with all groups significantly increasing response accuracy from pre- to post-test (all *p*s < 0.01) and from pre- to retention test (all *p*s < 0.01). Although performance slightly decreased from post- to retention test, this difference did not reach significance (all *p*s > 0.30). However, the test-related main effect was overlain by a significant group × test interaction, *F*(4,82) = 2.59, *p* = 0.04, $\eta_{p}^{2}$ = 0.11. Statistically, this interaction is explained by a virtually identical base level at pre-test for all three groups, *F*(2,44) = 0.01, *p* = 0.99, $\eta_{p}^{2}$ < 0.00, but learning differences with the largest improvement for the control group (pre-post: +36.8%, *p* < 0.01, $\eta_{p}^{2}$ = 0.79; pre-ret: 36.1%, *p* < 0.01, $\eta_{p}^{2}$ = 0.81), followed by the functional group (pre-post: +29.4%, *p* < 0.01, $\eta_{p}^{2}$ = 0.69; pre-ret: +27.9%, *p* < 0.01, $\eta_{p}^{2}$ = 0.68) and the dysfunctional group (pre-post: +22.2%, *p* < 0.01, $\eta_{p}^{2}$ = 0.68; pre-ret: +18.4%, *p* < 0.01, $\eta_{p}^{2}$ = 0.71). In more detail, in the post-test, *F*(2,44) = 3.53, *p* < 0.05, $\eta_{p}^{2}$ = 0.15, as well as in the retention test, *F*(2,44) = 5.25, *p* < 0.05, $\eta_{p}^{2}$ = 0.20, a significant main effect for group appeared with the control group, outperforming the dysfunctional group in both tests (all *p*s < 0.05, all $\eta_{p}^{2}$ > 0.21). In contrast, no differences were revealed between the functional and the control groups (all *p*s > 0.15, all $\eta_{p}^{2}$ < 0.07, all 1-β > 0.40) and between the functional and the dysfunctional groups (all *p*s > 0.08, all $\eta_{p}^{2}$ < 0.09, all 1-β > 0.34). Hence, the results show that (1) over the course of learning all groups improved response accuracy but (2) the functional showed no improvements when compared to the control group and (3) the functional cue group did not significantly differ from the dysfunctional group in post- and retention test.

### Discussion

In the context of perceptual training, the gaze-path-cue method is based on the assumptions that (a) gaze-path cueing leads to an outlasting change in gaze behavior, (b) in the direction of an instructed (and superior) gaze path, which (c), in turn, enhances decision-making as visual attention is shifted and crucial cues can be better processed due to the improved gaze strategy. Furthermore, it is expected that the application of the relatively costly gaze-path-cue method is outweighed by the learning advantages when being compared with simple instructions on trying to improve anticipatory skills. The main study provides insights regarding all these assumptions and expectations. In more detail:

(a) Based on the findings of Savelsbergh et al. (2010) that learners tend to exhibit a gaze pattern quite similar to the to-be-learned gaze path by themselves, it was decisive to include a dysfunctional group in the design of the main study. This is particularly true as, also in our study’s pre-test, participants used a gaze strategy that already corresponds well to the strategy observed in expert beach volleyball players (see **Figure 5**). As a consequence, such as the case in the Savelsbergh et al. (2010) study, opportunities for learning were limited anyway. However, it also must be recognized that the GPI score of the dysfunctional group shows a remarkable shift toward a dysfunctional gaze behavior directly after the intervention as well as in the 7-days-delayed retention test evidencing an outlasting change of the learners’ gaze behavior. Hence, overall, it could be confirmed that gaze-path-cueing affects gaze behavior not only on a short-term performance level – as it had been demonstrated in the preparatory study – but also on the long-term level of gaze-path learning. Above, the shortness of the intervention phase has to be emphasized as participants practized less than 1 h with overall 84 visual-cued trials only. Thus, it must be concluded that gaze-path cueing appears to be a very strong method for altering gaze behavior.

However, no learning effect was revealed for the functional group. Albeit, as already stated above, the opportunity of learning was reduced for the functional group in comparison with the dysfunctional group, it must be stressed that the average GPI difference of the functional group’s participants in both phases (P1: 3.6° of visual angle, P2: 2.6° of visual angle) clearly exceeded the area in which visual information can be acutely perceived (maximally 2° of visual angle, Hirsch and Curcio, 1989). Hence, on average, the participants definitely processed information that is different from the information in the cued areas so that the differences should allow both experimental groups to learn the respective cue path and to reduce the GPI over the course of learning.

The absence of learning in the functional group cannot be ascribed to methodological issues (cf. Abernethy et al., 2012) since exactly the same type of visual cue was used and the same methods of measurement and data analyses were applied as for the dysfunctional group. Consequently, the rate of learning may have been limited by the fact that most participants already followed a gaze path that was similar to that of the elite model (and presented during the cued training) (cf. Savelsbergh et al., 2010). This effect, in turn, might be attributed to a general incapability of the human perceptual system to detect differences between an actual gaze path and a to-be-learned gaze path highlighted by visual cues as soon as this difference falls below a certain threshold. Consequently, gaze-path cueing would be a strong method for beginners wherein huge gaze-related differences are present but would turn out to be of minor value for perceptual training if these differences are smaller or in case of advanced learners or experts.

In response to that issue, one could try to optimize the gaze-path-cue method by the introduction of additional information. If, as suggested before, the problem is caused by the incapability of detecting differences between the actual and the cued gaze path, it would be of particular interest to enrich the visual-cue technique by augmented-feedback routines. Although, compared to motor learning (e.g., Salmoni et al., 1984), little is known on the effectiveness of augmented feedback in perceptual learning, a small number of empirical studies gives rise to optimism at least. In this respect, advantages of providing feedback have been found for discrimination learning (for an overview, e.g., Trommershäuser et al., 2009), and Wilson et al. (2011) successfully introduced feedback routines in learning a laparoscopic skill by contrasting the learners’ gaze behavior to the behavior of an expert (although the feedback relevance was not explicitly tested). Hence, as a next step in gaze-path-cue research, it seems worthwhile to empirically study the effects of augmented-feedback routines on the perceptual-learning rate.

(b) An alternative explanation for the missing effects of gaze-path cueing in the functional group refers to the actual functionality of the to-be-learned gaze path. In more detail, this point regards two issues. The first one relates to the question whether an expert gaze path is distinguished by an overall superiority or whether, due to a level-specific fit of perception and action, an expert gaze path is functional for experts only. At this point, a fundamental problem of perceptual training in sports is addressed that seems to require a massive amount of further research.

When claiming, for the moment, that experts’ gaze behavior can also be used as a target value for the training of non-experts, the second issue arises, which refers to the problem of identifying a valid expert gaze path. In our study, the functional gaze path had been derived from expert interviews (Schläppi-Lienhard and Hossner, 2015) as well as from the, to our knowledge, most comprehensive data set on beach volleyball experts’ gaze behavior in the defence situation at hand (Hossner et al., in preparation). However, an a posteriori conducted analysis of the gaze patterns found in the main study gives rise to doubts regarding the actual functionality of the to-be-learned gaze paths. In more detail, for all three groups *post hoc* median splits were calculated with regard to the actual absolute distance to the functional gaze path, resulting in comparatively close and comparatively far trials. The analysis revealed no relevant differences in response accuracy between close and far trials for P1. However, in the pre-, post-, and retention tests of P2, participants of the functional and of the control groups showed higher response accuracies when their gaze was rather far from (*M* = 66.5%) than close to (*M* = 70.1%) the gaze path that had been regarded as functional. This implies that visually pursuing the cue in P2 rather degrades but does definitely not enhance decision-making. Consequently, it has to be considered that simply mimicking experts’ gaze paths might not provide the best way of applying the gaze-path-cue technique. Instead, it seems worthwhile to study whether highlighting certain cues at certain points in time leads to better learning results than a mere presentation of a to-be-learned gaze path. Regarding this question, again, further research is needed in the future.

(c) When it comes to the above-stated issue on the gaze-path-cue method inherent assumption that a change in gaze behavior might improve decision-making, the data at hand are not exactly cause for optimism. This judgment is based on the fact that (in contrast to Abernethy et al., 2012) all three groups showed significant improvements in response accuracy from pre- to post- and retention test (see **Figure 6**). This means that, in the first place, a gaze-path change in the direction of a dysfunctional pattern does not prohibit but it is (when compared to the control group) detrimental to anticipatory-skill learning. Above, a simple instruction to identify different attack patterns does not result in inferior learning than the application of the gaze-path-cue method. Finally, the improvements in the participants of the functional cue and, in particular, of the control groups occurred independently of any change in gaze behavior.

Consequently, one must infer that highlighting crucial visual information using markers rather occasionally distracts from the subjacent information source than enhancing its processing. In more general terms, instructing just to watch an area that is highlighted by a cue seems insufficient to extract the underlying information. Thus, with respect to the superior learning of the control group, there must have been changes in either the processing or in making use of the processed information for decision-making (see also Ryu et al., 2015). Neither the assumption that enhanced perception necessarily comes along with improved gaze behavior nor the assumption that gaze improvements necessarily enhance perception is supported by the results at hand.

Taken together, our findings may be understood as an emphasis of the – above cited – warning voiced by (Abernethy et al. (2012, p. 152) that, in perceptual training, gaze-path cueing must be used with caution. However, what also could be shown by the data is that gaze-path cueing is a strong technique for the induction of gaze changes on a short-time scale of immediate (preparatory study) as well as on a large-time scale of permanent changes (main study). For this reason, before completely denying any value of this method, it should be tested whether and to what degree the effects of a gaze-path-cue intervention could be increased by certain optimisations. As substantiated above, these optimisations may concern the application of effective cue markers, the addition of augmented feedback regarding the difference between actual and desired gaze path, and the use of spatio-temporal cueing patterns that, instead of simply pursuing an expert gaze path, highlight certain areas at certain points in time. Finally, a gaze-path-cue technique, which has been optimized in these respects, might also be a valuable add-on to other methods of perceptual training. Consequently, it should also be empirically tested whether, for instance, the effect of an instruction intervention (as in the main study’s control group) would be enhanced if the instructions were underlay with matching visual cues. All these questions are currently pursued by our research group.

## Conflict of Interest Statement

The authors declare that the research was conducted in the absence of any commercial or financial relationships that could be construed as a potential conflict of interest.

## APPENDIX

Instructions for the functional and dysfunctional groups. “In the following, we will show you videos with different beach-volleyball attacking scenes. In addition, the gaze strategy of the currently best female Swiss beach volleyball player – XX XX – will be displayed as red circular patches. In the following 12 block of 12 trails each, your task is to learn the gaze path of XX XX. Over the learning phase the frequency of cued scenes will be gradually reduced.”

Instructions for the control group. “In the following, we will show you videos with different beach-volleyball attacking scenes. In the following 12 block of 12 trails each, your task is to learn the different attacking strategies.”
